# The 20th International AIDS Conference: where do we go from here?

**DOI:** 10.7448/IAS.17.1.19331

**Published:** 2014-07-18

**Authors:** Mark A. Wainberg, Susan Kippax, Marlene Bras, Papa Salif Sow

**Affiliations:** 1 McGill University AIDS Centre, Jewish General Hospital, Montreal, QC, Canada; 2 Journal of the International AIDS Society, Geneva, Switzerland; 3 Social Policy Research Centre, University of New South Wales, Sydney, Australia; 4 Department of Infectious Diseases, University of Dakar, Dakar, Senegal

This year marks 10 years from the initiation of the *Journal of the International AIDS Society* (JIAS). *JIAS* was started as an attempt to take advantage of low-cost online publishing and to make it possible for scientists from developing countries to publish their findings in the scientific literature. During the past decade, we have published a wide array of manuscripts that provide testimony to the wonderful progress that has been made in regard to the HIV epidemic.

The HIV/AIDS epidemic is unprecedented for a number of different reasons. As HIV scientists from around the world gather in Melbourne for another International AIDS Conference, it behooves us to reflect on the progress that has been made and speculate on what needs to be accomplished over the next decade.

Who can remember another disease that arose as quickly as HIV did? In the early 1980s, millions of people around the world became infected by HIV, and almost all of them died due to the fact that antiretroviral (ARV) drugs had not yet been developed. Thankfully, the availability of safe and well-tolerated ARVs over most of the past 25 years has now resulted in a situation in which almost all infected persons who are fortunate enough to live in wealthy countries can aspire to live for many years. The reason for this is that HIV disease has been transformed into a chronic manageable condition in almost all high-income-country settings. Antiretroviral therapy (ART) has been successful in both wealthy countries and resource-limited settings, and ART has had significant impact on morbidity and mortality in sub-Saharan African countries with a high burden of HIV [1]. Furthermore, ART not only prolongs life but also dramatically reduces HIV transmission. ART is now available to 10 million people living with HIV in low- and middle-income countries. These achievements are a result of transformative science, advocacy, political commitment and effective partnerships with affected communities. However, substantial challenges still exist in regard to maintaining access to ART and finding the funding for lifelong ART for the more than 34 million people who are living with HIV.

For this and other reasons, there is now a widespread consensus that a cure for HIV disease may be the only truly effective way to deal over the long term with the HIV epidemic. Although the global programmes that exist to provide ARVs to people in low- and middle-income countries have largely been successful, they will probably be unsustainable over future decades for reasons due to the high costs that may be required, potentially exceeding hundreds of billions of dollars over the next 20 years. For this reason, many healthcare economists have proclaimed that high-income countries may not be able to provide this necessary assistance unless an unprecedented rebound takes place in regard to the global economy. It should also be recognized that it is not easy for patients to have to take drugs every day for the rest of their lives. Moreover, attempts to develop effective HIV vaccines have been largely unsuccessful despite the heroic efforts of the research community.

In concert with a search for a cure, strengthened HIV prevention efforts are also needed [2]. Mother-to-child transmission has been dramatically reduced, although programmes preventing mother-to-child transmission are not reaching all pregnant women. With regard to the sexual transmission of HIV, HIV prevention strategies such as condom use and a reduction of number of sexual partners need reinvigoration. As has been demonstrated, the provision of sterile needles and syringes is the most successful way of reducing HIV transmission among people who inject drugs: countries that do not provide such programmes should be urged to do so. In the absence of a cure, the best public policy response is to invest more in prevention [3]. However, funding for prevention is declining in many countries, and HIV prevention is faltering [4]. Although HIV prevention was successful in the first 20 years of the epidemic, infection rates are on the rise again in some countries, both high-income as well as low- and middle-income countries [5].

Medical male circumcision is proving to be effective in reducing HIV transmission to men in generalized epidemic settings. Efforts are also underway to protect against new HIV infections through the use of ARVs that are administered on a prophylactic basis to people at risk for acquisition of HIV. These programmes are referred to as pre-exposure prophylaxis (PrEP) [6], and several studies suggest that PrEP may be able to protect as many as 50% of individuals at risk from acquisition of HIV, so long as they take their ARVs in a fully adherent manner. Although the CAPRISA 004 clinical trial provided encouraging results, more research is needed to evaluate the efficacy of vaginal microbicides that contain an ARV [7].

PrEP has also stimulated a related area of research termed “treatment as prevention” (TasP). The concept in this case is that the successful mass use of ARVs will lead to diminished viral loads within populations and that the transmission of new HIV infections will be greatly diminished, if not eliminated [8]. However, concerns have been raised that the development of HIV drug resistance and the transmission of drug-resistant viruses might thwart such efforts. This notwithstanding, hope is also provided by the recent development of novel ARVs that may not be as prone to drug resistance as earlier drugs.

It is therefore ironic that there is now unprecedented optimism: optimism in regard to stemming HIV infections and optimism in regard to potential curative strategies for HIV [9,10]. This is in spite of the faltering of HIV prevention and, with regard to a cure, in spite of knowledge of the problems involved and the recognition that HIV can establish latent long-lived cellular reservoirs that cannot be easily targeted by either currently available ARVs or immunological strategies [11]. A large number of approaches are now aimed at the reactivation of these reservoirs, such that latently infected cells may be effectively targeted by more traditional ARV treatments [12]. Both public and private granting agencies in many developed countries have now established dedicated funding programmes that seek to attain the eradication of HIV infection. Indeed, a large critical mass of scientists are fully engaged in this effort, and the goal of finding a cure for HIV has been highlighted at many recent HIV conferences as it will again at the 20th International AIDS Conference to take place in Melbourne during July 2014.

We hope that at least some of the thousands of scientists in attendance at the Melbourne conference will produce efforts that will lead us to the goal of finding a cure and more effective prevention. The success of current ARV usage in the treatment of HIV has the potential to provide benefits to both society as a whole and the individuals receiving therapy. People who are successfully treated with ARVs have vastly diminished viral loads in their bodies and, as a consequence, are far less able to transmit HIV than are untreated individuals [13,14]. Indeed, the use of ARVs at a population level has led to significant reductions in community viral load, which refers to the average viral burden in the community of persons living with HIV, in one particular geographic area in South Africa [15]. While this finding demonstrates evidence of the impact of ART on HIV acquisition, it is possible that ART may have less impact in reducing new HIV infections in countries or among populations where very high levels of ART coverage have been reached – such as in Europe and Australia [16]. Indeed, there have been increases, instead of the expected decreases, in HIV incidence in gay communities in many high-income countries where HIV testing rates and uptake of ARVs are high [17]. Risk compensation and associated decrease in condom use by gay men appear to be part of the problem.

The progress in the fight against HIV/AIDS has been dramatic and substantial. Few would have predicted the advances that have taken place over the past quarter century, and we do have a right to be more optimistic today than at any time since the start of the HIV epidemic. However, multiple problems remain, and these include a wide array of social issues pertaining to HIV infection, such as stigmatization of persons living with HIV, criminalization of transmission of HIV infection, a continuation of high-risk behaviour and demonization of homosexual activity by certain political leaders, among others. In spite of all the problems, however, we are closer than ever before to finding the clues that will ultimately end the HIV epidemic. *JIAS* hopes to continue to publish the ground-breaking research that will help to lead us to this goal.
